# SREBP2 promotes macrophage alternative activation and allergic airway inflammation independent of cholesterol biosynthesis

**DOI:** 10.1038/s41419-026-08910-y

**Published:** 2026-06-03

**Authors:** Yinfang Wu, Kua Zheng, LingLing Dong, Shenwei Gao, Yanping Wu, Zhengyuan Liu, Jieyu Li, Haipin Chen, Qingyu Weng, Miao Li, Chen Zhu, Jiahuan Xu, Songmin Ying, Zhihua Chen, Wen Li

**Affiliations:** 1 Key Laboratory of Respiratory Disease of Zhejiang Province, Department of Respiratory and Critical Care Medicine, Second Affiliated Hospital of Zhejiang University School of Medicine, Hangzhou, Zhejiang China; 2 Department of Pharmacy, Center for Regeneration and Aging Medicine, the Fourth Affiliated Hospital of School of Medicine, and International School of Medicine, International Institutes of Medicine, Yiwu, China

**Keywords:** Monocytes and macrophages, Asthma, Mechanisms of disease, Inflammation

## Abstract

Immunometabolic reprogramming is increasingly recognized as a critical regulator of macrophage activation and function. This study aimed to elucidate the role of the SREBP2-cholesterol biosynthetic pathway in M2 activation of interstitial macrophages (IMs) during allergic asthma. We observed a significant expansion of M2-polarized IMs with heightened SREBP2-cholesterol biosynthetic activity in the lungs of asthmatic mice. IL4, a key pro-allergic cytokine in allergic asthma, was found to induce SREBP2 maturation and activate the cholesterol biosynthetic signaling pathway in macrophages. Moreover, inhibiting SREBP2 maturation restrains IL4-induced M2 activation, both in vitro and in vivo. Intriguingly, this effect was independent of intracellular cholesterol levels; instead, cholesterol itself negatively regulated SREBP2 maturation and M2 activation. Mechanistically, mature SREBP2 transcriptional modulated IRF4 expression and interacted with it to promote IL4-induced M2 activation. Additionally, myeloid-specific *Scap* deficiency impeded the SREBP2 maturation process in macrophages, leading to reduced M2 activation and allergic airway inflammation. These findings highlight the pivotal role of the SREBP2-IRF4 axis in modulating M2 polarization of IMs and suggest potential therapeutic targets for asthma treatment.

## Introduction

Tissue-resident macrophages are essential for maintaining tissue homeostasis and serve as immune sentinels. They exhibit diverse functions, including phagocytosis of dead cells and pathogens, secretion of pro-inflammatory substances and growth factors, and remodeling of the extracellular matrix [1]. Macrophages can be activated by type-2 cytokines and differentiate into alternatively activated (M2) macrophages. These M2 macrophages can promote type-2 immune response and mediate wound healing, tissue repair, and immune modulation [2].

In allergic asthma, interstitial macrophages (IMs) can be activated by interleukin (IL) 4 and IL13, prompting their differentiation into M2-polarized IMs [2], while alveolar macrophages (AMs) are less responsive than IMs [3]. These M2-polarized IMs serve as the primary effector cells in allergic asthma, promoting type-2 immune response [4]. It is well-established that their activation status is intrinsically linked to metabolic remodeling [5], which includes increased consumption of fatty acids and glucose, as well as enhanced mitochondrial respiratory capacity. Nevertheless, little is known about the impact of cholesterol metabolism on the activation and function of M2-polarized IMs.

Cholesterol biosynthesis represents a significant part of cellular cholesterol metabolism, and nearly all cells possess the capability to synthesize cholesterol [6, 7]. Cholesterol biosynthesis is mainly regulated by sterol regulatory element-binding protein 2 (SREBP2) [6, 7], which primarily promotes the transcription of genes encoding enzymes involved in cholesterol biosynthesis. It is synthesized as endoplasmic reticulum (ER)-anchored inactive precursors, which interact with SREBP-cleavage activating protein (SCAP). SREBP2 undergoes a canonical SCAP-, Site-1 protease (S1P), Site-2 protease (S2P)-dependent pathway to becoming active, and this process is so-called SREBP2 maturation. To be specific, SCAP escorts the SREBP2 precursor to translocate from the ER to the Golgi apparatus, where the SREBP2 precursor is processed by S1P- and S2P-mediated two steps of proteolytic cleavage to become a nuclear active form and finally mature SREBP2 entry into the nucleus to regulate gene transcription [8]. Recent work has also implied that SREBP2 promotes tumor necrosis factor (TNF)-induced inflammatory macrophage activation [9]. Yet, the precise role of the SREBP2-cholesterol biosynthetic pathway in M2 macrophage activation and the pathogenesis of allergic asthma remains to be elucidated.

Here, we utilized single-cell RNA sequencing (scRNA-seq) to systematically characterize the expansion of M2-polarized IMs in the lungs of asthmatic mice. Comparative transcriptomic profiling uncovered the dysregulation of the SREBP2-cholesterol biosynthetic pathway in M2-polarized IMs. We further investigated the functions and detailed molecular mechanisms of the SREBP2-cholesterol biosynthetic pathway in macrophage activation and allergic airway inflammation.

## Results

### Asthmatic mice exhibit elevated M2-polarized IMs with augmented SREBP2-cholesterol biosynthesis pathway activity in pulmonary tissues

**Fig. 1:**
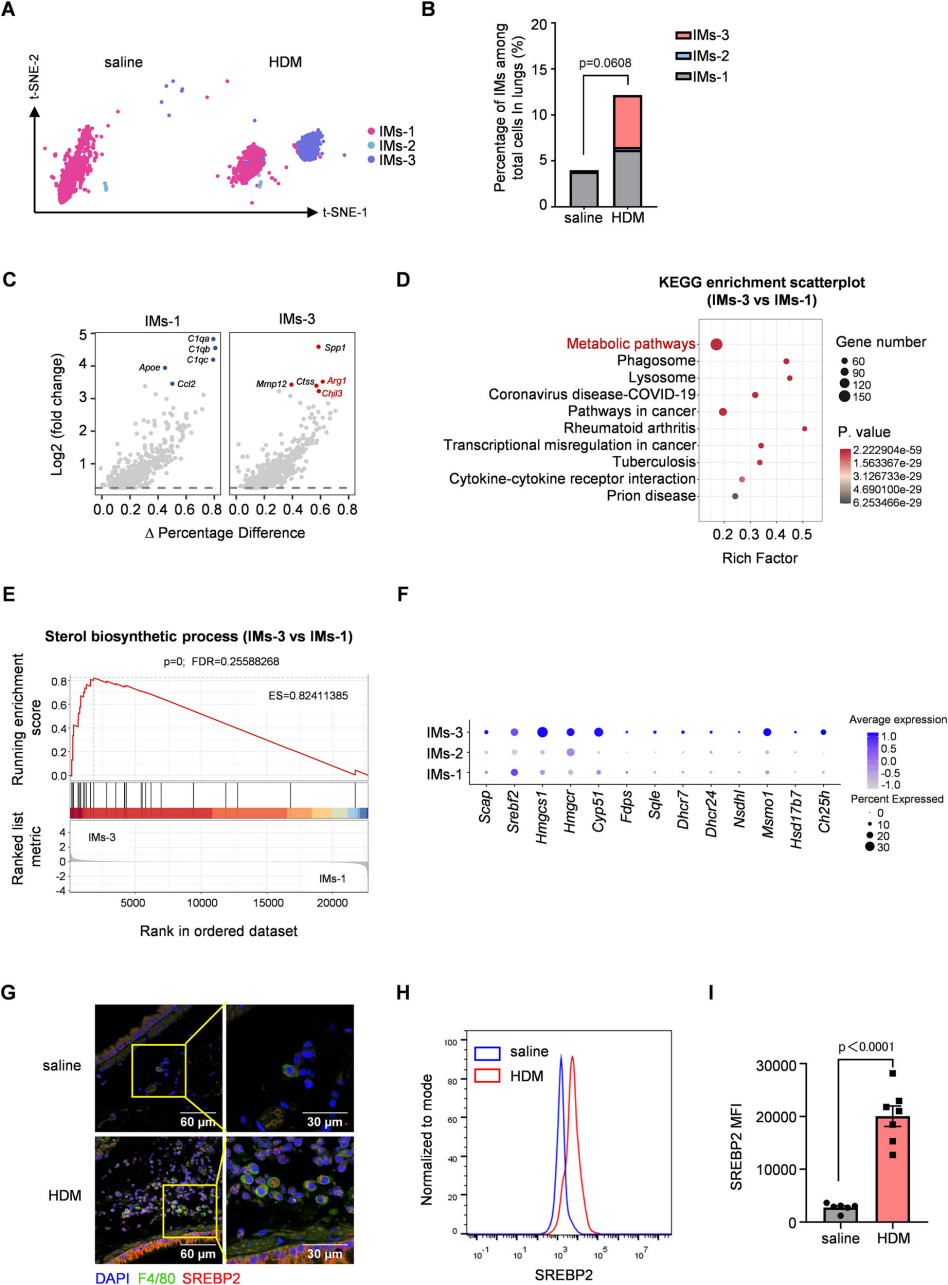
Asthmatic mice exhibit elevated M2-polarized IMs with augmented SREBP2-cholesterol biosynthesis pathway activity in pulmonary tissues. An HDM-induced allergic asthma model was established, and lung tissues from HDM-treated and control mice were collected for scRNA-seq analysis (*n* = 3 per group). **A** t-SNE visualization of IMs subpopulations. **B** Proportions of IMs-1, IMs-2, and IMs-3 in the saline group and HDM group. **C** Marker gene analysis for IMs-1 and IMs-3. **D** KEGG pathway enrichment analysis of differentially expressed genes in IMs-3 versus IMs-1. **E** Gene set enrichment analysis (GSEA) for sterol biosynthesis process-related gene signatures. **F** Expression levels of genes related to the SREBP2-cholesterol biosynthetic pathway in IMs-1, IMs-2, and IMs-3. **G** Confocal microscopy images showing SREBP2 (red), F4/80 (green), and DAPI (blue) staining in lung sections from saline-treated and HDM-treated mice (*n* = 5 per group). **H**, **I** Flow cytometry analysis of SREBP2 expression in IMs with semi-quantitative analysis (*n* = 6–7 per group). Data are presented as mean ± SEM; statistical analyses performed using Student’s t-test.

To delineate the heterogeneity of IMs in allergic asthma, we established a house dust mite (HDM)-induced murine asthma model and performed scRNA-seq on lung tissues, with saline-treated mice serving as controls. t-SNE analysis identified three distinct IM subsets: IMs-1, IMs-2, and IMs-3 (Fig. 1A). Under homeostatic conditions, IMs-1 constituted the predominant population, whereas HDM-challenged lungs exhibited a significant expansion of IMs-3, which was rarely detected in controls (Fig. 1A, B). Notably, IMs-3 displayed robust expression of M2 markers (*Arg1*, *Chil3*), suggesting a polarization shift toward an M2 phenotype in asthmatic lungs (Fig. 1C).

Differential gene expression analysis revealed substantial metabolic reprogramming in IMs-3 compared to IMs-1 (Fig. 1D), characterized by marked upregulation of the sterol biosynthesis pathway (Fig. 1E, F). Immunofluorescence co-staining of F4/80 and SREBP2 further confirmed enhanced SREBP2 maturation in IMs from asthmatic lungs (Fig. 1G). Additionally, flow cytometry analyses yielded consistent results (Fig. 1H, I), thereby suggesting the involvement of the SREBP2-cholesterol biosynthetic pathway in M2 activation of IMs during allergic airway inflammation.

### Allergic cytokine IL4 induces SREBP2 maturation and activates the cholesterol biosynthetic pathway in macrophages

**Fig. 2:**
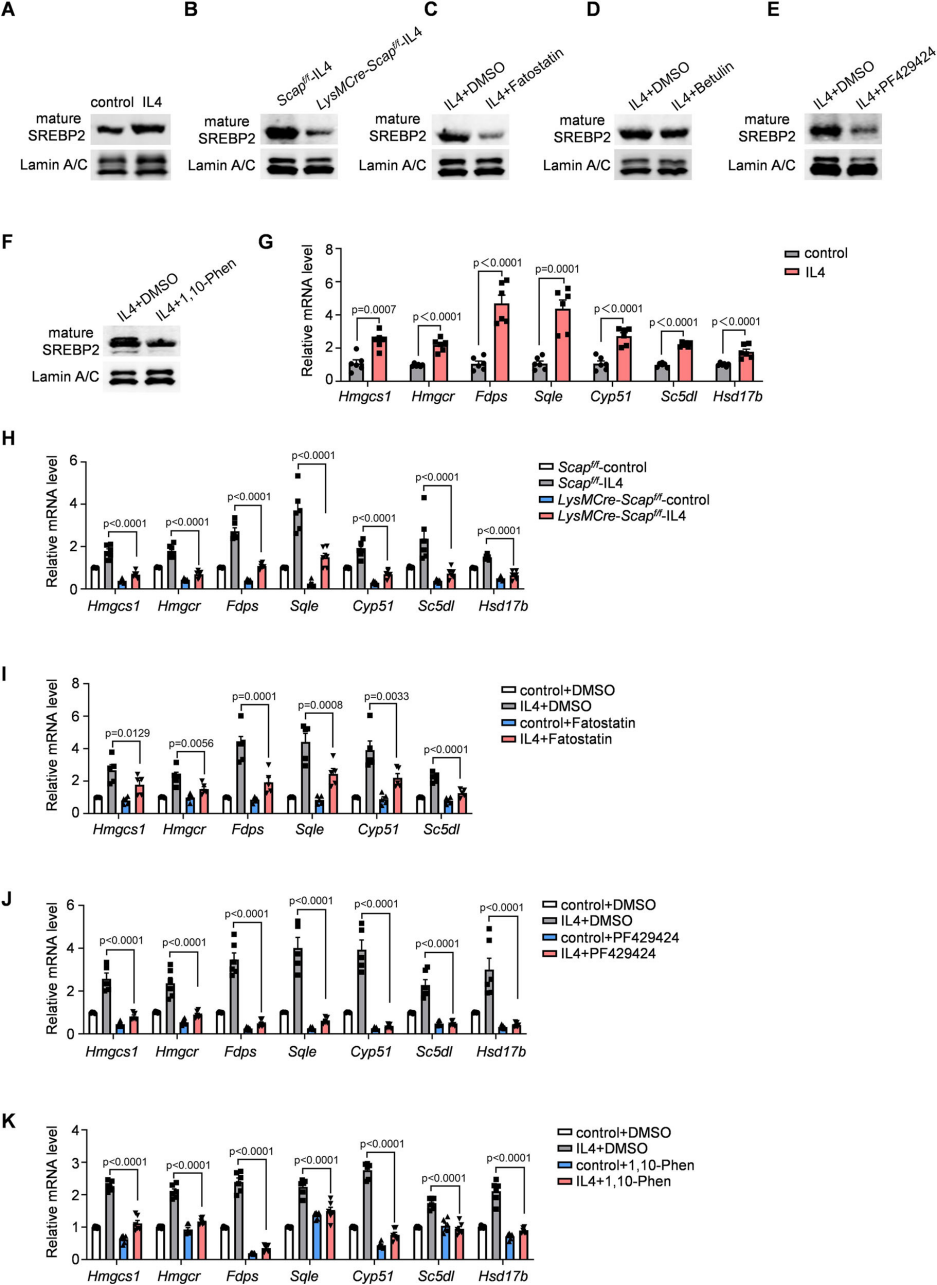
Allergic cytokine IL4 induces SREBP2 maturation and activates the cholesterol biosynthetic pathway in macrophages. **A** Immunoblotting of mature SREBP2 in BMDMs exposed to IL4 (100 ng/ml) for 24 h. **B** Immunoblotting of mature SREBP2 in IL4-treated BMDMs from *Scap*^*f/f*^ and LysMCre*-Scap*^*f/f*^ mice. **C**–**F** BMDMs were treated with 10 μM Fatostatin (**C**), or 10 μM Betulin (**D**), or 50 μM PF429424 (**E**), or 50 μM 1,10-Phen (**F**) together with IL4 (100 ng/ml) for 24 h, and the expression of mature SREBP2 was determined by western blotting. **G** Q-PCR for the expression of certain SREBP2-target genes in BMDMs stimulated with IL4 (100 ng/ml) for 24 h. **H** Q-PCR for the expression of certain SREBP2-target genes in BMDMs from *Scap*^*f/f*^ and LysMCre*-Scap*^*f/f*^ mice treated with IL4 (100 ng/ml) for 24 h. **I**–**K** BMDMs were treated with 10 μM Fatostatin (**I**), or 50 μM PF429424 (**J**), or 50 μM 1,10-Phen (**K**) together with IL4 (100 ng/ml) for 24 h, and the cells were harvested to detect the expression of certain SREBP2-target genes by Q-PCR. Data are representative of three independent experiments and presented as mean ± SEM; Differences between the two groups were identified using the Student’s t-test (**G**) and multiple groups (**H**–**K**) using one-way ANOVA.

We then evaluated the potential of allergic cytokines to regulate SREBP2 maturation and the cholesterol biosynthetic pathway in macrophages in vitro. Given the well-documented ability of IL4, a classical allergic cytokine, to induce M2 activation of IMs in allergic asthma, we exposed bone marrow-derived macrophages (BMDMs) to IL4. We found that IL4 stimulation significantly increased nuclear localization of the activated form of SREBP2 (mature SREBP2) (Fig. 2A). To investigate whether IL4-induced SREBP2 maturation occurred through the canonical SCAP-, S1P, S2P-dependent pathway, we used a combination of genetic and pharmacologic loss-of-function approaches. Mice with myeloid-specific deficiency of *Scap*, whose deletion renders the SREBP2 inactive precursor unable to translocate from ER to Golgi [7], were generated by crossing *Scap*^*f/f*^ mice with LysM-Cre mice. *Scap*^*−/−*^ BMDMs exhibited decreased levels of mature SREBP2 induced by IL4 (Fig. 2B). Fatostatin and betulin, two small molecules that block SREBP2 maturation by interacting with SCAP [10, 11], both prevented IL4-induced SREBP2 maturation (Fig. 2C, D). Then we tested whether S1P and S2P could contribute to this IL4-induced SREBP2 maturation. As expected, IL4-induced SREBP2 maturation was diminished when together with S1P inhibitor PF429424 [12] or S2P inhibitor 1,10-Phenanthroline (1,10-Phen) [13] (Fig. 2E, F). These data indicated that IL4 promoted SREBP2 maturation through the canonical pathway, i.e., the precursor was translocated from ER to Golgi and subsequently processed by S1P and S2P.

Mature SREBP2 mediates the expression of the genes associated with cholesterol biosynthesis [7], and we then found that IL4 increased the expression of SREBP2 target genes, including *3-hydroxy-3-methylglutaryl-coenzyme A synthase 1* (*Hmgcs1)*, *3-hydroxy-3-methylglutaryl-Coenzyme A reductase* (*Hmgcr)*, *farnesyl diphosphate synthetase* (*Fdps)*, *squalene epoxidase* (*Sqle)*, *cytochrome P450, family 51* (*Cyp51)*, *sterol-C5-desaturase* (*Sc5dl)*, and *hydroxysteroid (17-beta) dehydrogenase (Hsd17b)* (Fig. 2G), and either *Scap* deficiency or Fatostatin blocked the upregulation of these genes (Fig. 2H, I). Furthermore, inhibition of S1P or S2P exerted similar suppressive effects (Fig. 2J, K).

These data demonstrated that the allergic cytokine IL4 could induce SREBP2 maturation and substantially activate the cholesterol biosynthetic pathway in macrophages.

### SREBP2 maturation is crucial for M2 activation

**Fig. 3:**
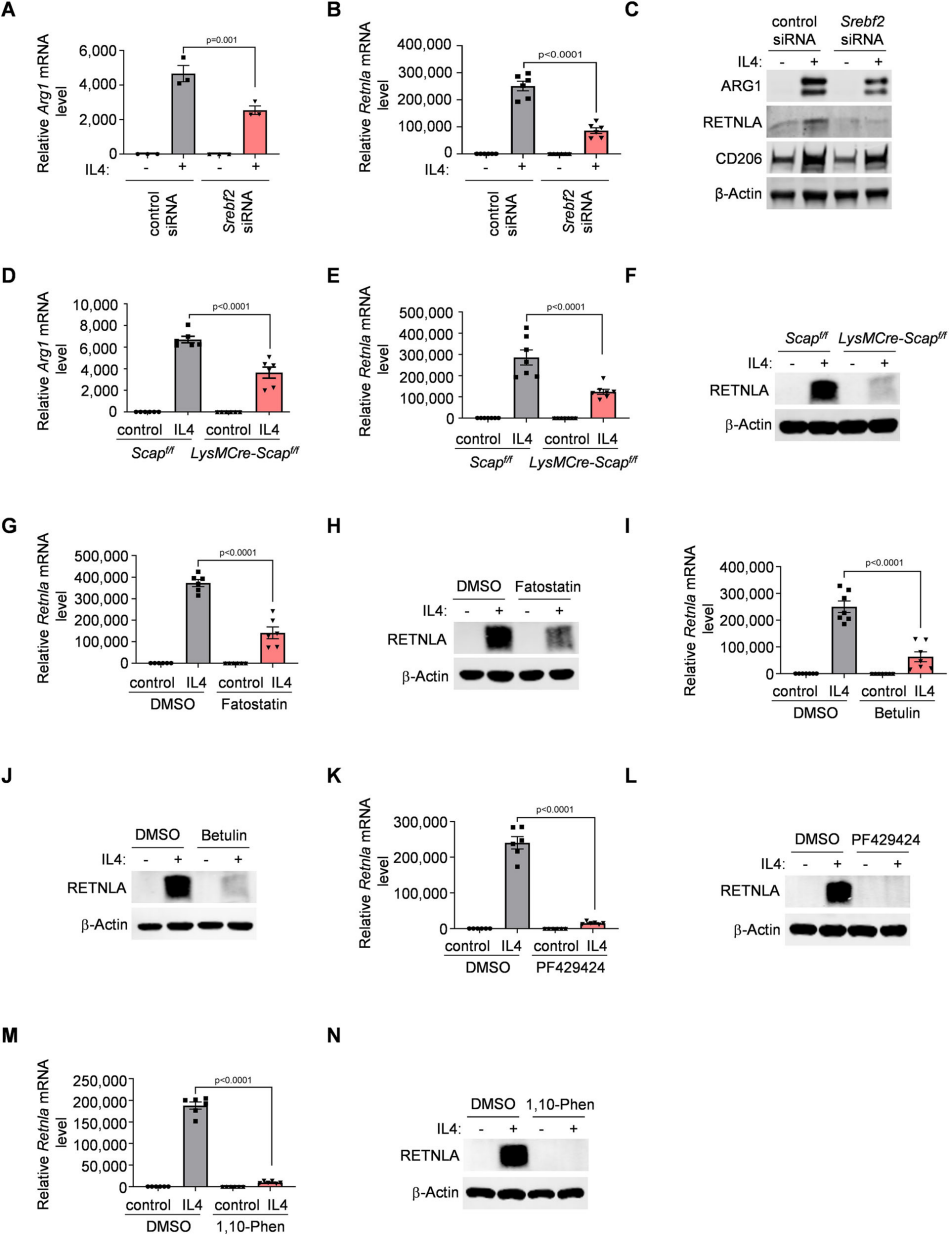
SREBP2 maturation is crucial for M2 activation. **A**–**C** BMDMs were transfected with *Srebf2* siRNA for 24 h, then treated with IL4 (100 ng/ml) for an additional 24 h, and cell extracts were collected for analysis of ARG1, RETNLA, and CD206 expression by Q-PCR and western blotting. **D-F** Q-PCR for the expression of *Arg1* (**D**) and *Retnla* (**E**) and immunoblotting of RETNLA (**F**) in IL4-treated BMDMs from *Scap*^*f/f*^ and LysMCre*-Scap*^*f/f*^ mice. **G**–**N** BMDMs were treated with 10 μM Fatostatin (**G**, **H**), or 10 μM Betulin (**I**, **J**), or 50 μM PF429424 (**K**, **L**), or 50 μM 1,10-Phen (**M**, **N**) together with IL4 (100 ng/ml) for 24 h, the relative mRNA levels of *Retnla* were measured by Q-PCR (**G**, **I**, **K**, **M**), while the protein levels of RETNLA were detected by western blotting (**H**, **J**, **L**, **N**). Data are representative of three independent experiments and presented as mean ± SEM; Differences were identified using one-way ANOVA.

Subsequently, we aimed to investigate the possible functions of SREBP2 maturation in IL4-induced M2 activation. First, we examined whether M2 activation was affected by the absence of SREBP2. We observed that silencing SREBP2 using siRNA targeting *sterol regulatory element-binding transcription factor 2* (*Srebf2)*, the gene encoding SREBP2, significantly inhibited the IL4-induced upregulation of M2 activation markers, including arginase 1 (ARG1), resistin-like molecule alpha (RETNLA), and CD206 (Fig. 3A–C). The knockdown efficiency of all siRNAs used in this study was shown in Fig. S1.

Then, we examined whether SCAP-SREBP2 ER-to-Golgi translocation was necessary for M2 activation. Notably, *Scap*^*−/−*^ BMDMs showed a remarkable decline in the expression of ARG1 and RETNLA (Fig. 3D–F). Consistent with this, Fatostatin and betulin, two widely used SCAP inhibitors, further confirmed the function of this process in M2 activation, which markedly inhibited the IL4-induced expression of RETNLA (Fig. 3G–J). These data indicated that SCAP-SREBP2 ER-to-Golgi translocation is essential for M2 activation.

We have shown in Fig. 2 that IL4 induced-SREBP2 maturation relied on the two-step cleavage by S1P and S2P, raising the possibility that S1P and S2P could also be involved in M2 activation. We then examined the role of S1P and S2P in M2 activation. As predicted, S1P or S2P inhibition substantially reduced the expression of RETNLA induced by IL4 (Fig. 3K–N).

Taken together, our data demonstrated that SREBP2 maturation was critically involved in IL4-induced M2 activation.

### Mature SREBP2 facilitates M2 activation independently of cholesterol levels, while cholesterol modulates M2 activation predominantly through its feedback regulation of SREBP2 maturation

**Fig. 4:**
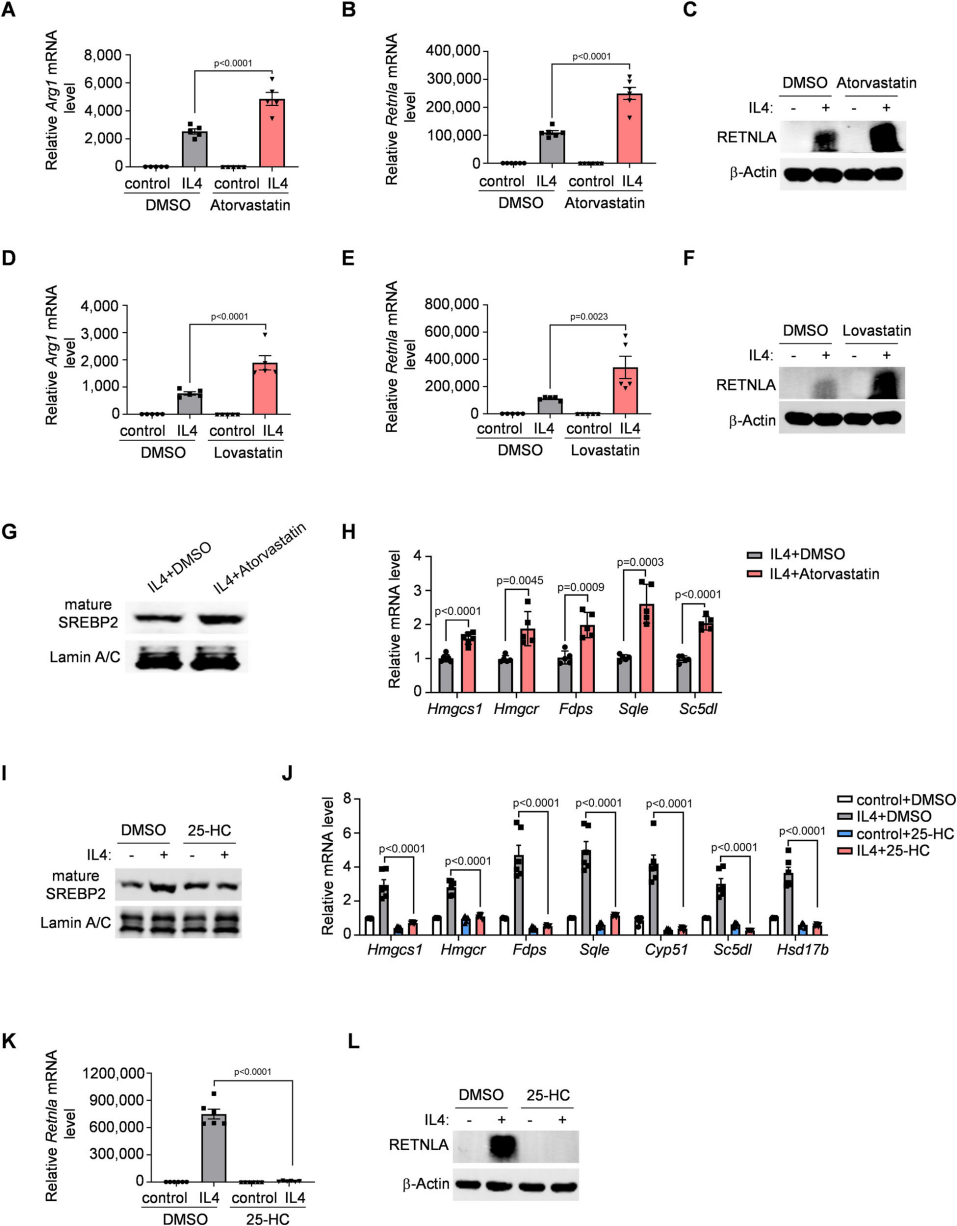
Mature SREBP2 facilitates M2 activation independently of cholesterol levels, while cholesterol modulates M2 activation predominantly through its feedback regulation of SREBP2 maturation. **A**–**F** BMDMs were treated with 10 μM Atorvastatin (**A**–**C**) or Lovastatin (**D**–**F**) together with IL4 (100 ng/ml) for 24 h, the relative mRNA levels of *Arg1* (**A**, **D**) and *Retnla* (**B**, **E**) were measured by Q-PCR, while the protein levels of RETNLA were detected by western blotting (**C, F**). **G**–**J** BMDMs were treated with 10 μM Atorvastatin (**G**, **H**), or 10 μM 25-HC together with IL4 (100 ng/ml) for 24 h, and the expression of mature SREBP2 was determined by western blotting (**G**, **I**). The expression of certain SREBP2-target genes was measured by Q-PCR (**H**, **J**). **K**, **L** RETNLA expression in IL4-treated BMDMs stimulated with 25-HC was determined by Q-PCR (**K**) and western blotting (**L**). Data are representative of three independent experiments and presented as mean ± SEM; Differences were identified using one-way ANOVA.

IL4 promoted SREBP2 maturation and subsequently induced the expression of genes encoding enzymes in the cholesterol biosynthesis pathway (Fig. 2). We next questioned whether the promotional effect of SREBP2 maturation on the M2 activation was associated with a disruption in cellular cholesterol homeostasis. Surprisingly, neither IL4 nor inhibition of IL4-induced SREBP2 maturation significantly altered the cellular cholesterol levels (Fig. S2). To further confirm the role of cholesterol in M2 activation, we used atorvastatin, which suppresses the de *novo* biosynthesis of cholesterol via inhibiting HMGCR, a rate-limiting enzyme in cholesterol biosynthesis. Intriguingly, atorvastatin effectively strengthened, rather than suppressed, M2 activation upon IL4 treatment in BMDMs (Fig. 4A–C). We also treated BMDMs with another inhibitor of HMGCR, lovastatin, and similar results were observed (Fig. 4D–F).

We proposed that atorvastatin and lovastatin inhibited the de *novo* biosynthesis of cholesterol, which in turn increased the activity of endogenous SREBP2. Indeed, we found that atorvastatin promoted the IL4-induced SREBP2 maturation (Fig. 4G) and upregulated the expression of certain SREBP2-target genes (Fig. 4H). Thus, the promotional effect of atorvastatin on M2 activation was likely due to the increased SREBP2 maturation.

To further clarify the role of cholesterol in SREBP2 maturation and M2 activation, we used 25-HC, an endogenous sterol. We found that 25-HC prevented IL4-induced SREBP2 maturation (Fig. 4I), decreased those measured SREBP2-target genes (Fig. 4J), and subsequently led to a reduction in the IL4-induced expression of RETNLA (Fig. 4K, L).

These findings suggested that mature SREBP2 facilitated M2 activation independently of cholesterol levels. Conversely, cholesterol appeared to modulate M2 activation predominantly through its feedback regulation of SREBP2 maturation.

### Mature SREBP2 controls M2 activation by interacting with IRF4 physically and functionally

**Fig. 5:**
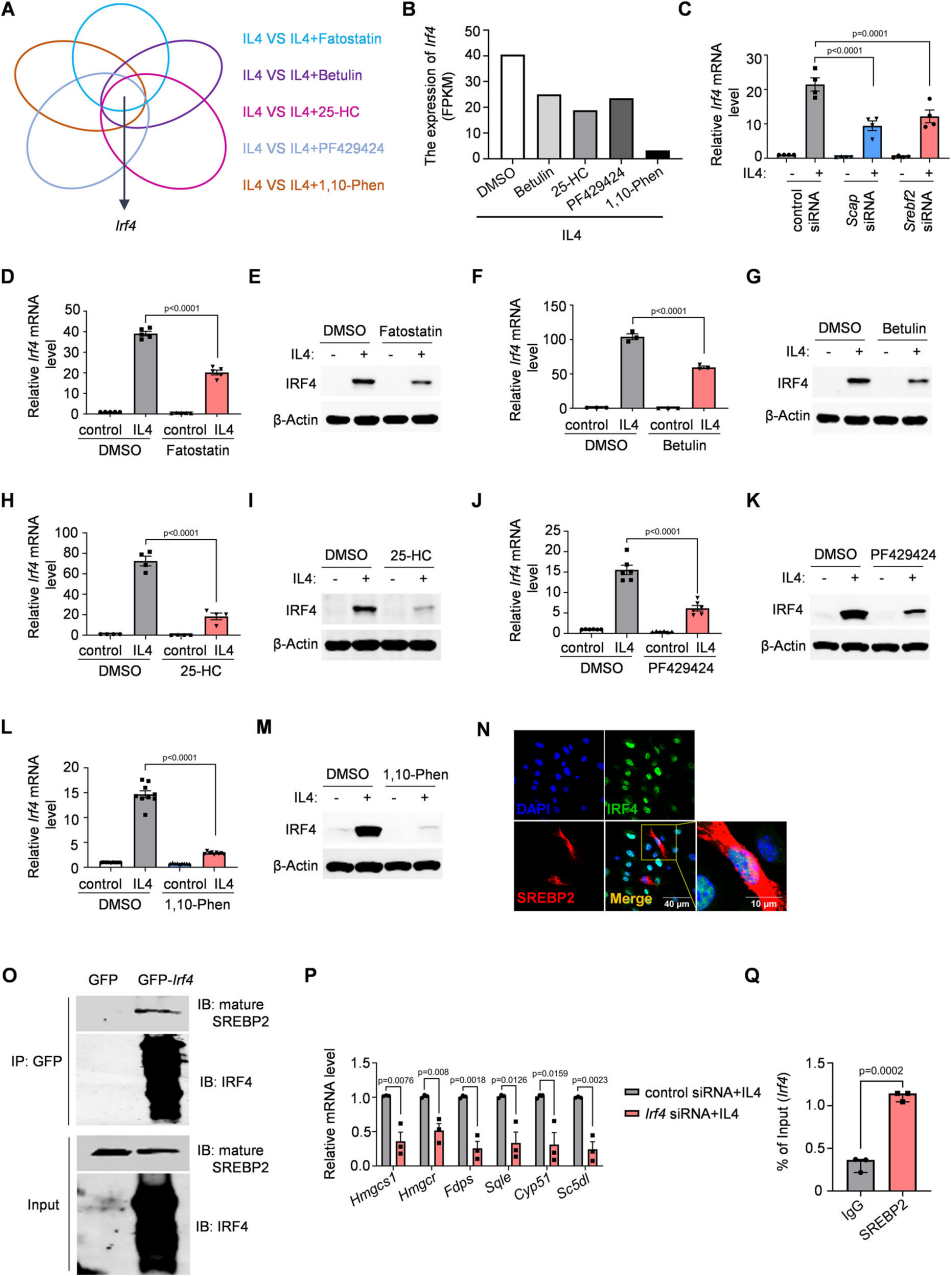
Mature SREBP2 transcriptional modulates IRF4 expression and interactes with it to promote M2 activation. **A**, **B** RNA-seq analysis for the expression of *Irf4* in IL4-treated BMDMs stimulated with 10 μM Betulin, or 10 μM 25-HC, or 50 μM PF429424, or 50 μM 1,10-Phen for 24 h. **C** BMDMs were transfected with *Srebf2* or *Scap* siRNA for 24 h, then treated with IL4 (100 ng/ml) for an additional 24 h, and the cell extracts were harvested for measuring the expression of *Retnla* by Q-PCR. **D**–**M** BMDMs were treated with 10 μM Fatostatin (**D**, **E**), or 10 μM Betulin (**F**, **G**), or 10 μM 25-HC (**H, I**), or 50 μM PF429424 (**J**, **K**), or 50 μM 1,10-Phen (**L**, **M**) together with IL4 (100 ng/ml) for 24 h, the relative mRNA levels of *Irf4* were measured by Q-PCR (**D**, **F**, **H**, **J**, **L**), while the protein levels of IRF4 were detected by western blotting (**E**, **G**, **I**, **K**, **M**). **N** IL4-treated BMDMs were analyzed for co-localization of IRF4 with SREBP2 by confocal microscopic imaging. **O** HEK293T cells were transfected with GFP-*Irf4* and control GFP plasmid for 72 h, and GFP immunoprecipitates were analyzed for IRF4 and mature SREBP2 by western blotting. **P** BMDMs were transfected with *Irf4* siRNA for 24 h, then treated with IL4 (100 ng/ml) for an additional 24 h, and cell extracts were collected for detection of canonical SREBP2-target gene expression by Q-PCR. **Q** ChIP analysis of the occupancy of *Irf4* promoter in mature SREBP2-overexpressing THP-1 cells. Data are representative of three independent experiments and presented as mean ± SEM; Differences between the two groups were identified using the Student’s t-test (**P**, **Q**) and multiple groups (**C**, **D**, **F**, **H**, **J**, **L**) using one-way ANOVA.

Through RNA sequencing analysis (RNA-seq), we found the expression of *Irf4* in common downregulated in M2 macrophages when SREBP2 maturation was inhibited with betulin, 25-HC, PF429424, or 1,10-Phen (Fig. 5A, B), raising the possibility that IRF4 might be the downstream regulator of the SREBP2 signaling pathway.

Previous works have shown that IRF4 is implicated in macrophage M2 activation [14, 15]. In keeping with this, we also observed that IL4 stimulation significantly increased both mRNA and protein expression of IRF4 (Fig. S3A–D), and deletion of *Irf4* resulted in decreased M2 activation (Figure S3E). Then, we tested whether SREBP2 maturation controlled M2 activation via IRF4. We found that IL4-induced expression of *Irf4* was greatly diminished in the absence of *Scap* or *Srebf2* (Fig. 5C) or during the inhibition of SCAP-SREBP2 ER-to-Golgi translocation by Fatostatin (Fig. 5D, E), or Betulin (Fig. 5F, G), or 25-HC (Fig. 5H, I), or by PF429424 or 1,10-phen which inhibited SREBP2 cleavage in Golgi (Fig. 5J–M). Thus, these data altogether supported that mature SREBP2 was required for the expression of IRF4 in M2 macrophages.

We next sought to elucidate how mature SREBP2 regulates the expression of IRF4. We observed that IRF4 was predominantly located in the nucleus (Figure S3C). Given that mature SREBP2 was the nuclear active form of SREBP2, we investiaged whether mature SREBP2 and IRF4 could interact physically. As expected, immunofluorescence analysis revealed partial colocalization of mature SREBP2 with IRF4 (Fig. 5N), suggesting potential proximity. Of note, there is a phenomenon, considered as a form of feed-forward regulation, in which a transcription factor promotes its coactivator expression and then interacts with that activator to induce downstream gene expression [16, 17]. Consistent with this paradigm, we demonstrated that mature SREBP2 promoted IL4-induced IRF4 expression (Fig. 5C–M). Furthermore, coimmunoprecipitation assays revealed a strong interaction between mature SREBP2 and IRF4 (Fig. 5O), and knockdown of *Irf4* attenuated the expression of canonical SREBP2-target genes (Fig. 5P), indicating that SREBP2 upregulates IRF4 and cooperates with it to mediate downstream gene transcription. Since SREBP2 is known to regulate the transcription of both cholesterol biosynthesis genes and inflammatory genes, we wondered whether IRF4 was a direct transcriptional target of SREBP2. Chromatin immunoprecipitation (ChIP) analysis confirmed that mature SREBP2 directly bound to the *Irf4* promoter (Fig. 5Q and Fig. S4), supporting transcriptional regulation of IRF4 by SREBP2.

Taken together, these findings demonstrated that mature SREBP2 transcriptional modulated IRF4 expression and interacted with it to promote IL4-induced M2 activation.

### Myeloid-specific Scap deficiency attenuates HDM-induced M2 activation and allergic airway inflammation

**Fig. 6:**
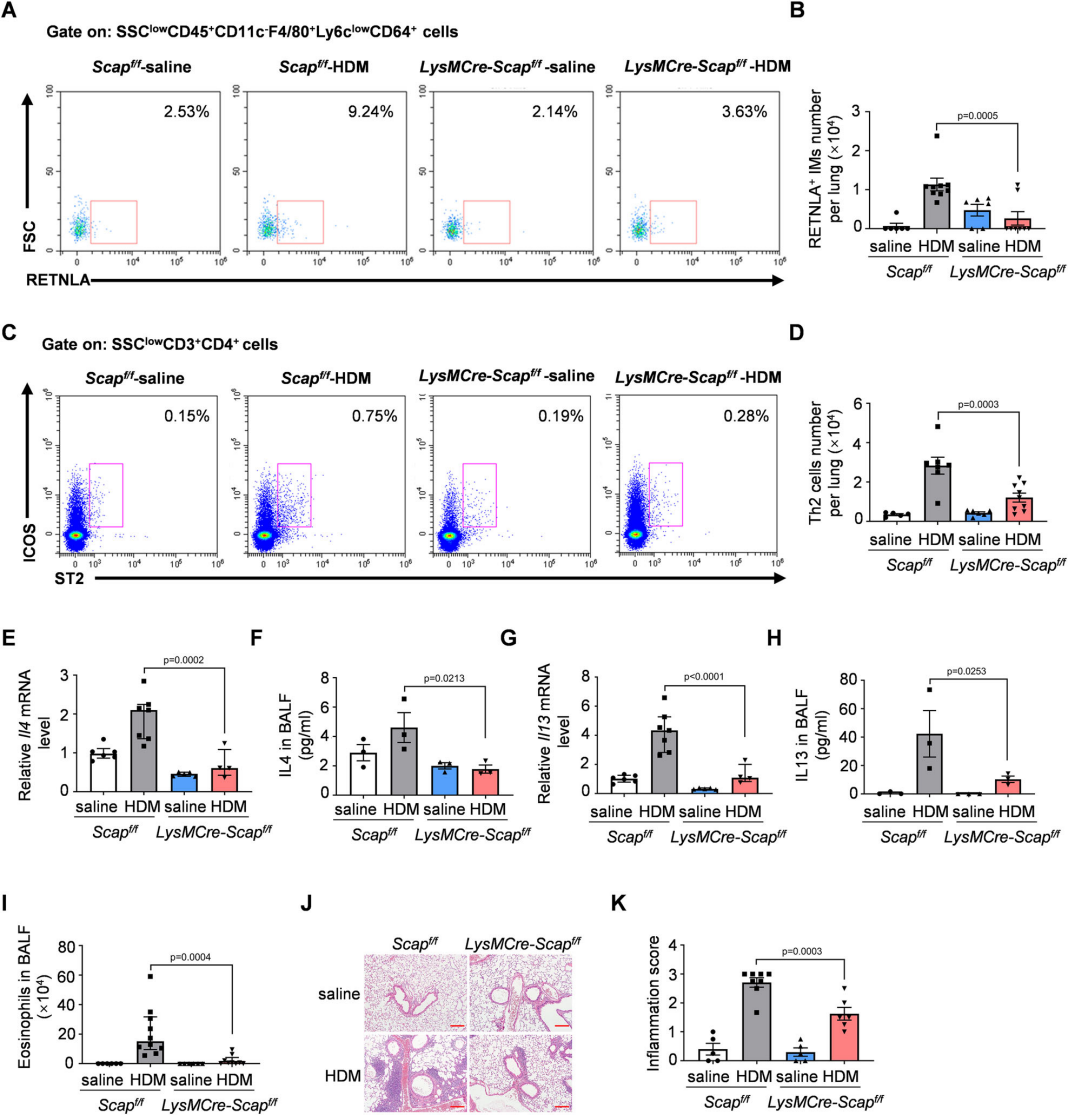
Myeloid-specific *Scap* deficiency attenuates HDM-induced M2 activation and allergic airway inflammation. *Scap*^*f/f*^ and LysMCre*-Scap*^*f/f*^ mice were administered intratracheally with HDM to induce allergic airway inflammation. 72 h after the last HDM exposure, **A**–**D** M2 macrophages (**A**, **B**) and Th2 cells (**C**, **D**) were detected by FACs analysis (*n* = 5–9 per group). **E**–**H** The relative mRNA level of *Il4* (**E**) and *Il13* (**G**) in the lungs was measured by Q-PCR, and the secretion of IL4 (**F**) and IL13 (**H**) in the BALF supernatants was determined by ELISA (*n* = 3–7 per group). **I** The number of eosinophils in the BALF was assessed (*n* = 6–10 per group). **J, K** Representative images (**J**) and semi-quantification (**K**) of lung sections stained with H&E (*n* = 5–8 per group, scale bar: 200 μm). Data are presented as mean ± SEM; Differences were identified using one-way ANOVA.

In allergic asthma, M2-polarized IMs mainly display a pro-inflammatory role [4]. We then assess the function of macrophage-localized SREBP2 maturation in M2 activation and HDM-induced allergic airway inflammation in vivo by using mice with myeloid-specific deficiency of *Scap* (LysMCre-*Scap*^*f/f*^), whose deletion could stop SREBP2 maturation in macrophages as shown in Fig. 2B. According to the published work, hyperresponsiveness to IL4 was a feature of lung IMs, and AMs are less responsive than IMs during allergic airway inflammation [3]. Therefore, lungs were isolated and stained with IMs markers (Fig. S5A) to further confirm the role of *Scap* deficiency in M2 activation in vivo. As expected, the number of RETNLA^+^ IMs was higher in the lungs of HDM-induced asthmatic mice than in saline-treated control mice, and this upregulation effect was largely diminished in the absence of *Scap* in macrophages (Fig. 6A, B).

Considering the well-established effect of M2 macrophages in type-2 response, we examined whether *Scap* deficiency affected the HDM-induced Th2 response in allergic asthma. Th2 cells were defined as live SSC^low^CD3^+^CD4^+^ICOS^+^ST2^+^ cells (Fig. S5B). As predicted, there were lower numbers of Th2 cells in the lungs (Fig. 6C, D), as well as less production of IL4 and IL13 both in the lungs and BALF of LysMCre-*Scap*^*f/f*^ asthmatic mice (Fig. 6E–H).

As a consequence, LysMCre-*Scap*^*f/f*^ mice developed less severe eosinophilic airway inflammation than wild-type (*Scap*^*f/f*^) mice after HDM administration, as revealed by the decreased eosinophils in bronchoalveolar lavage fluid (BALF) (Fig. 6I) and around the airways (Fig. 6J, K).

Together, these results showed that myeloid-specific *Scap* deficiency negatively regulated M2 activation of IMs, suppressed Th2 immune response, and ultimately contributed to attenuated allergic airway inflammation.

## Discussion

Immunometabolic reprogramming is increasingly recognized as a critical regulator of macrophage activation and function [18, 19]. In this study, we integrated cholesterol metabolism with M2-polarized IMs, uncovering a previously unrecognized role of the SREBP2-cholesterol biosynthetic pathway in M2 activation of IMs during allergic asthma. We observed a significant expansion of M2-polarized IMs with heightened SREBP2-cholesterol biosynthesis activity in the lungs of asthmatic mice. IL4, a key pro-allergic cytokine in allergic asthma, was found to induce SREBP2 maturation and activate the cholesterol biosynthetic signaling pathway in macrophages. Moreover, inhibiting SREBP2 maturation restrains IL4-induced M2 activation, both in vitro and in vivo. Intriguingly, this effect was independent of intracellular cholesterol levels; instead, cholesterol itself negatively regulated SREBP2 maturation and M2 activation. Mechanistically, mature SREBP2 transcriptional modulated IRF4 expression and interacted with it to promote IL4-induced M2 activation. The physiological relevance of these findings was confirmed by the marked attenuation of HDM-induced allergic airway inflammation in mice with myeloid-specific *Scap* deficiency, highlighting the therapeutic potential of targeting this pathway.

The relationship between SREBP2 and cholesterol is a two-way process. SREBP2 is well-known as a crucial player in the cholesterol biosynthetic pathway [7, 8], by acting as a master transcriptional regulator of the expression of cholesterol biosynthesis-associated genes. On the other hand, cholesterol can also negatively regulate the activation of SREBP2 via an ER-located delicate feedback system, which senses the declined or excess cholesterol to activate or inactivate the SREBP2 maturation [20, 21]. Thus, it raises the possibility that the role of SREBP2 in inflammatory responses may be either dependent or independent of cholesterol. There are several studies have indicated that SREBP2 exerts its immunomodulatory effects through its ability to control cholesterol biosynthesis. For example, reports have identified that SREBP2 activation in intestinal epithelial cells increases blood and liver cholesterol, and overexpression of intestinal SREBP2 further enhances diet-induced hepatic inflammation and fibrosis via increased hepatic cholesterol [22, 23]. Moreover, a recent report has demonstrated that P53-mediated tumor suppression correlates with the reduced maturation of SREBP2, the expression of cholesterol biosynthetic genes, and cholesterol and its metabolic intermediates. On the contrary, increasing evidence reveals that SREBP2 may function without affecting cholesterol homeostasis. A recent study has reported that SCAP-SREBP2 promotes NLRP3 inflammasome activation, and this promoting effect is independent of the cellular level of cholesterol [11]. Another recent study has also indicated that SREBP2 promotes inflammation mainly in a direct manner by binding to inflammatory and interferon response target genes [9]. Of note, both studies emphasize that although the cholesterol biosynthetic pathway is tightly coupled to NLRP3 inflammasome activation or TNF-induced inflammatory macrophage polarization, the pro-inflammatory effect is independent of cholesterol. Instead, cholesterol displays an opposite function toward SREBP2, most likely due to its feedback regulation of SREBP2 maturation [9, 11]. Additionally, a very recent study has reported a similar result [24]. In line with these observations, our findings strongly supported the idea that SREBP2 promoted M2 activation without affecting cholesterol homeostasis and further confirmed that changes in the cellular level of cholesterol negatively influenced M2 activation, most likely via its regulation of SREBP2 maturation. However, it should be noted in our study that IL4 increased the expression of cholesterol biosynthetic genes while failing to alter the cellular level of cholesterol. Given that the cellular cholesterol level reflects the dynamic balance between biosynthesis, uptake, export, and storage, one plausible explanation is that IL4 might also activate the process of export or storage to ensure cholesterol homeostasis.

Canonically, SREBP2 functions as a transcriptional factor, which is not only critical for the control of cellular cholesterol homeostasis-associated gene expression [7] but also required for the transcription of inflammatory genes [9, 24]. For instance, a recent study has identified that carnosic acid decreases the cholesterol level by inhibiting SREBP2 activity and the expression of its target genes involved in cholesterol biosynthesis, including *Hmgcs, Hmgcr, Fpps*, and so on [25]. SREBP2 has also been reported to have the ability to directly bind and activate inflammatory target genes in TNF-stimulated macrophages [9]. Moreover, SREBP2 can transactivate miRNA-92a to increase endothelial innate immunity in response to oxidative stress [26]. However, a recent study has identified a totally different manner in which SCAP-SREBP2 binds to the NLR family, pyrin domain containing 3 (NLRP3) to form a ternary complex and escorts the NLRP3 to translocate from the ER to the Golgi apparatus for the assembly and activation of the inflammasome [11]. Thus, these results indicate that SREBP2 may play critical immunomodulatory roles in various ways. To support this, our results pointed out a new manner of regulation for SREBP2, in which SREBP2 transcriptional modulated IRF4 expression and interacted with it to promote IL4-induced M2 activation.

It is noteworthy that SREBP2 is a promising yet challenging therapeutic target due to its dual roles in inflammation and systemic lipid homeostasis [27]. Systemic inhibition may attenuate inflammation but risks disrupting essential cholesterol synthesis, potentially triggering compensatory pathways that further diminish efficacy and induce metabolic imbalance. Critically, SREBP2 also functions in a cell type–specific manner: while its suppression in immune cells may dampen inflammatory responses [11], inhibition in hepatocytes could aggravate hepatic steatosis [28]. Therefore, future therapeutic strategies should prioritize cell-selective modulation or tissue-targeted delivery systems to precisely regulate SREBP2 activity in pathological contexts while preserving its vital physiological functions, thereby facilitating safer and more effective clinical translation.

IRF4, a member of the interferon regulatory factor family, is not only a master regulator of immune cell differentiation [29] but also a central and essential node of the gene regulatory network to control distinct B- and T-cell fates [30]. Besides, an increasing body of evidence shows that IRF4 plays a vital role in macrophage alternative activation [14, 15, 31–34]. Recently, IRF4 has been reported to integrate metabolic reprogramming with immunomodulatory roles in macrophages. For example, a recent report has identified that IRF4 was crucial for MTOR-mediated regulation of glycolysis in M2 macrophages [31], providing a newly detailed mechanism for regulating IRF4 in M2 activation. In our study, we further found that SREBP2 transcriptional modulated IRF4 expression and interacted with it to regulate IL4-induced M2 activation, suggesting a functional link between cholesterol metabolism and IRF4 signaling.

The role of M2 macrophages in allergic airway inflammation remains a debatable topic [35, 36]. M2 macrophages are protective by mediating allergen clearance through mannose receptor C type-1 (MRC1) [37] and suppressing eosinophilic inflammation and Th2 activation through Interleukin 1 receptor antagonist (IL1RA) [38]. Conversely, M2 macrophages may also function deleteriously through their ability to secrete CCL17 and CCL22 to recruit Th2 cells to enter the lung and secrete transforming growth factor, beta 1 (TGFβ1), to induce airway remodeling [35]. Besides, the M2 marker arg1 can stimulate bronchoconstriction and airway remodeling, and another M2 marker Retnla can also cause airway remodeling [35]. In our study, we found that deficiency in myeloid-specific *Scap* resulted in decreased M2 macrophages and Th2 cells in the lung, which eventually contributed to the attenuated HDM-induced allergic airway inflammation, supporting that M2 macrophages played a harmful role in allergic airway inflammation.

This study has the following limitations: (1) Clinically accessible samples from asthma patients—such as peripheral blood, bronchoalveolar lavage fluid, induced sputum, or bronchial brush samples—do not contain lung IMs. Direct assessment of SREBP2 activity in human IMs requires scRNA-seq or cell sorting analyses using lung tissue from asthma patients, which is extremely difficult to obtain in clinical practice; 2) The study primarily relies on mouse macrophage models, whereas human macrophages exhibit a limited response to classical M2-polarizing stimuli in vitro, limiting the functional validation of the SREBP2-IRF4 axis in human cells.

In conclusion, our study establishes a novel immunometabolic paradigm in the pathogenesis of allergic asthma, demonstrating that the SREBP2-IRF4 axis plays a critical role in regulating M2 activation of IMs, and that cholesterol modulates this process likely in a feedback manner. This study deepens our understanding of how cholesterol metabolism regulates M2 activation and offers new opportunities for therapeutically modulating macrophage biology in allergic airway inflammation.

## Materials and Methods

### Mice

C57BL/6 mice were purchased from Slaccas Animal Center (Shanghai, China). *Scap*^*f/f*^ mice were kindly provided by Prof. Bao-Liang Song. They were respectively crossed with LysMCre mice to generate myeloid cell-specific *Scap* knockout (LysMCre-*Scap*^*f/f*^) mice. Age- and sex-matched *Scap*^*f/f*^ mice were used as the control. All mice were on the background of C57BL/6. Primers used for genotyping are listed in Table S1. Animals were allocated to experimental groups using a randomization method. Animal care and experimental protocols were approved by the Ethical Committee for Animal Studies at Zhejiang University.

### Cells

Mouse BMDMs were generated as described previously [39]. Briefly, bone marrow cells were isolated and cultured with DMEM containing 10% (v/v) FBS and 20 ng/ml mouse M-CSF for 5 days to obtain BMDMs. HEK-293T cells were purchased from the American Type Culture Collection and were cultured in DMEM containing 15% (v/v) FBS. To generate a stable cell line overexpressing the mature form of human SREBP2, THP-1 cells were transduced with a lentiviral construct encoding a truncated SREBP2 fragment (amino acids 1–481), referred to as mature SREBP2, fused with a 3×GS linker and an EGFP tag. An empty lentiviral vector served as the control. Cells were transduced at an optimized multiplicity of infection (MOI). At 48 h post-transduction, puromycin selection was initiated to enrich a polyclonal population with stable genomic integration. Successful expression of the mature SREBP2-EGFP fusion protein was verified by fluorescence microscopy, and elevated mature SREBP2 protein levels were confirmed by Western blotting. The resulting stable cell line was subsequently used in ChIP assays.

### Reagents

Antibodies used in this study were listed below: anti-ARG1 (16001-1-AP) was from Proteintech; anti-IRF4 (15106) were from Cell Signaling Technology; anti-SREBP2 (ab30682) and anti-RETNLA (ab39626) were from Abcam; anti-Lamin A/C (sc-20681), anti-β-Actin (sc-47778), and anti-SREBP2 (sc-13552) were from Santa Cruz Biotechnology; anti-CD206 (24595) and anti-SREBP2 (25940) were from Cell Signaling Technology; anti-CD16/CD32 (156603), anti-CD45 (103138), anti-F4/80 (123118), anti-ICOS (313508), anti-ST2 (146605), and anti-CD64 (139313) were from Biolegend; anti-CD11c (11-0114-82), anti-CD4 (11-0041-81), and anti-RETNLA (12-5441-82), and anti-Ly6c (45-5932-82) were from eBioscience; anti-CD3 (562600) were from BD Pharmingen.

Recombinant murine IL-4 (PeproTech, 214-14) was purchased from PeproTech. The following compounds were obtained from Selleck Chemicals: Fatostatin (S8284), PF429242 (S6418), and lovastatin (S2061). Betulin (B9757), 25-HC (H1015), 1,10-phenanthroline (131377), and atorvastatin (PZ0001) were sourced from Sigma-Aldrich. All primers used in this study were synthesized by Sangon Biotech.

A set of siRNAs of control (sc-37007), *Scap* (sc-36463), *Srebf2* (sc-36560), and *Irf4* (sc-35713) was purchased from Santa Cruz Biotechnology.

The Mouse IL4 ELISA kit (431107) was purchased from Biolegend, and the Mouse IL13 ELISA kit (BMS6015) was purchased from eBioscience.

### Generation of HDM-induced allergic airway inflammation model

As previously described, mice were administered intratracheally with 100 μg HDM on days 0, 7, and 14. Then, mice were sacrificed on day 17 for inflammation and FACs analysis.

### Mouse scRNA-seq sample preparation, library construction, and analysis

Lung tissues were dissected and mechanically dissociated into small fragments (~0.5 mm^2^) in chilled PBS (RNase-free, Ca²⁺/Mg²⁺-free) before enzymatic digestion. Tissue digestion was performed at 37 °C for 20 min using an enzyme cocktail containing 0.35% collagenase IV, 2 mg/ml papain, and 120 U/ml DNase I. The reaction was neutralized with cold 10% FBS/PBS, and the cell suspension was sequentially filtered through 70 µm strainers. After centrifugation (300 g, 5 min, 4 °C), erythrocytes were lysed with ACK lysis buffer. After dead cells were removed, cell viability (> 90%) was assessed using propidium iodide exclusion, and the concentration was adjusted to 700–1200 cells/µl for 10x Genomics Chromium Single Cell library preparation.

Sequencing libraries were generated following the 10x Genomics protocol, and paired-end sequencing (150 bp) was performed on an Illumina NovaSeq 6000 platform (LC-Bio, Hangzhou) with a minimum sequencing depth of 20,000 reads per cell.

Sequencing output files were demultiplexed and converted to FASTQ format using Illumina’s bcl2fastq software (version 2.20). Initial data processing, including sample demultiplexing, barcode processing, and unique molecular identifier (UMI) counting, was performed using the Cell Ranger (10X Genomics). Our analysis encompassed 77,979 single cells isolated from six murine lung specimens (3 saline-treated controls and 3 HDM-treated samples) using the 10X Genomics Chromium Single Cell 3’ Solution. Following initial processing, we implemented stringent quality control measures: removal of genes detected in fewer than 3 cells, exclusion of cells with fewer than 500 detected genes, elimination of cells with UMI counts less than 500, and removal of cells exhibiting mitochondrial gene content exceeding 25%. These quality filters resulted in a final dataset of 63,275 high-quality cells for downstream analysis. Subsequent computational analysis was performed using Seurat (version 4.1.1) and included: data normalization, PCA dimensionality reduction, shared nearest-neighbor graph-based clustering, and t-SNE visualization. Differential gene expression analysis between clusters was conducted using the Wilcoxon rank-sum test with the following stringent criteria: minimum percentage of cells expressing the gene > 10%, average log2 fold-change > 0.26, and adjusted *p*-value ≤ 0.01.

Since all samples were processed simultaneously under consistent experimental conditions, no obvious batch effect was observed among the 6 samples; therefore, batch-effect correction was not applied.

### BALF collection and analysis

Briefly, mice were sacrificed 72 h after the last exposure to HDM. Then, 0.7 ml PBS was lavaged into the lungs and withdrawn twice to collect BALF. The BALF cells were used for the total number and differential counts, and the remaining BALF supernatants were used for cytokine analysis.

### Hematoxylin & eosin (H&E) analysis

Mice were sacrificed 72 h after the last exposure to HDM, and the lungs were removed and prepared as paraformaldehyde-fixed, paraffin-embedded lung sections for H&E analysis. Semi-quantification was performed according to the published work [40].

### siRNA transfection

BMDMs (1 × 10^6^ cells) were transfected with 90 nM siRNA using the transfection reagent (Thermo Fisher Scientific, 13778150) following the manufacturer’s instructions.

### Plasmid transfection and coimmunoprecipitation

HER-293T cells were transfected with GFP-*Irf4* plasmid using PloyJet in vitro DNA Transfection reagent (SignaGen Laboratories, SL100688) according to the manufacturer’s protocol. 72 h after transfection, cells were harvested and lysed for coimmunoprecipitation analysis. IRF4 protein was immunoprecipitated with anti-GFP beads (Chromotek, gta-20), and the co-IP samples were analyzed for the expression of IRF4 and SREBP2 by western blotting.

### Quantitative PCR

The total RNA was extracted using RNAiso Plus (Takara Bio, 9109) and then reverse-transcribed into cDNA using reverse transcription reagents (Takara Bio, DRR037A). Quantitative PCR was performed using SYBR Green Master Mix (Takara Biotechnology, DRR041A) on a StepOne real-time PCR system (Applied Biosystems) to quantify gene expression. Primers for quantitative PCR are listed in Table S2.

### Western blot assay

BMDMs were lysed with RIPA buffer containing protease inhibitor (Roche Diagnostic GmbH, 04-693-116-001). The lysates were separated by SDS-PAGE and immunoblotted with the relevant antibodies using standard protocols.

### ChIP

ChIP assays were performed using a commercial ChIP kit (Thermo Scientific, AB408973) according to the manufacturer’s instructions. Briefly, approximately 1 × 10⁷ cells (mature SREBP2-overexpressing THP-1 cells) per sample were cross-linked with 1% formaldehyde for 10 min at room temperature. Then, cells were washed twice with ice-cold PBS containing protease inhibitors, lysed, and subjected to sonication on ice to shear chromatin into fragments, with a total sonication duration of 30 min. The sonicated lysates were divided into three groups: one incubated overnight at 4 °C with an anti-SREBP2 antibody (Cell Signaling Technology, 25940), another with a control IgG, and the third retained as an input control. Following immunoprecipitation, immune complexes were captured, washed, eluted, and reverse cross-linked in accordance with the kit protocol. DNA was purified and analyzed by quantitative PCR using primers specific to the IRF4 promoter region. Primer sequences were as follows: Forward: 5′-AGTTGCAGGTTGACCTACGG-3′; Reverse: 5′-TTCACCCGTTGAGCTTTCGA-3′. Enrichment was calculated as a percentage of input DNA and subjected to statistical analysis.

### RNA-Seq analysis

RNA isolation was performed using RNAiso Plus (Takara Bio, 9109) according to the manufacturer’s guidelines. RNA integrity was evaluated using an Agilent 2100 Bioanalyzer (Agilent Technologies, Palo Alto, CA, USA). Qualified RNA samples were subsequently processed for library preparation using Illumina Novaseq6000 by Gene Denovo Biotechnology Co., Ltd (Guangzhou, China).

### Total cholesterol assessment

We examined the total cholesterol levels in BMDMs using a commercial cholesterol detection kit (Thermo Scientific, A12216). Briefly, BMDMs were lysed in an equal volume of RIPA buffer, and 50 μL of cell lysate was incubated with 50 μL of Amplex Red cholesterol detection reagent for 30 min at 37 °C. Fluorescence intensity was measured using a Varioskan Flash Spectral Scanner (Thermo Scientific, Waltham, MA, USA). Cholesterol concentrations were determined using the equation generated using the same commercial kit: cholesterol concentration = 0.006 × fluorescence intensity − 0.6551. All measured cholesterol concentrations fell within the kit’s specified sensitivity and linear detection range. To account for variations in sample loading, total cholesterol levels were normalized to the protein content in each lysate. Specifically, the ratio of total cholesterol to protein content was calculated for each sample, with the control group ratio set to 1. The resulting normalized values were used for statistical analysis.

### Immunofluorescence staining

The expression of IRF4 was assessed by immunofluorescence staining as described before [41]. BMDMs were fixed and stained with anti-IRF4 (1:1000) overnight at 4 °C. Fluorescence pictures were photographed with a confocal microscope. Image J software was used for semi-quantification, and the results were shown as the relative fluorescence intensity normalized to the control.

### ELISA

The concentrations of IL4 and IL13 in BALF supernatants were measured using ELISA kits according to the manufacturer’s instructions.

### Flow cytometry

Once mice were sacrificed, the lungs were collected and digested with the solution of type I collagenase (Gibco, 17100017) at 37 °C for 60 min to obtain lung mononuclear cells. The total number was counted with an automatic cell counter (Countstar, IC1000). Then, equal numbers of cells were used for Th2 cells (live SSC^low^CD3^+^CD4^+^ ICOS^+^ST2^+^) or IMs (live SSC^low^CD45^+^CD11c^−^F4/80^+^Ly6c^−^CD64^+^) staining according to the previous studies [42, 43]. The Cytoflex flow cytometry system (Beckman Coulter) was applied to flow cytometry analysis.

### Statistical analysis

The results are presented as mean ± SEM. Statistical analysis was carried out by GraphPad Prism 8 (version 8.4.2, GraphPad Software). Between-group comparisons were analyzed by unpaired two-tailed Student’s t-test for two groups or one-way ANOVA with Sidak’s multiple comparisons test for multiple groups.

## Supplementary information

Supplementary materials

Original Western blots

## Data Availability

RNA-seq results are available in the Gene Expression Omnibus database under accession code (GSE188356). Other data are available from the corresponding authors upon request.
