# Supplementary material for: SREBP2 promotes macrophage alternative activation and allergic airway inflammation independent of cholesterol biosynthesis
Source: Cell Death Dis. 2026 Jun 3;17(1):697. doi: 10.1038/s41419-026-08910-y (PMC13448065; doi:10.1038/s41419-026-08910-y)
Supplement: Supplementary file 1 [file 41419_2026_8910_MOESM1_ESM.docx]

**Supplementary Materials**

**Supplementary figures and legends**

**Figure S1**

**Fig. S1 | siRNA knockout efficiency. A-C** BMDMs were transfected with *Scap* (**A**), or *Srebf2* (**B**), or *Irf4* (**C**) siRNA for 24 h, then the cell extracts were harvested for knockout efficiency analysis. Data are representative of three independent experiments and presented as mean ± SEM; statistical analyses performed using Student's t-test.

**Figure S2**

**Fig. S2 | Neither IL4 nor inhibition of IL4-induced SREBP2 maturation alters the cellular levels of cholesterol. A** BMDMs were harvested 1, 8, 24 h after IL4 (100 ng/ml) exposure and analyzed for the concentration of cholesterol. **B-E** BMDMs were treated with 10 μM Fatostatin (**B**), or 10 μM Betulin (**C**), or 50 μM PF429424 (**D**), or 50 μM 1,10-Phen (**E**) together with IL4 (100 ng/ml) for 24 h, the level of cholesterol was determined by the cholesterol detection kit. Data are representative of three independent experiments and presented as mean ± SEM.

**Figure S3**

**Fig. S3 | IRF4 is required for IL4-induced M2 activation.** BMDMs were stimulated with IL4 (100 ng/ml) for 24 h. **A** The relative mRNA level of *Irf4* was measured by Q-PCR. **B** The protein level of IRF4 was detected by western blotting. **C, D** Representative immunofluorescence images (**C**) and semi-quantification of IRF4 (**D**). **E** BMDMs were transfected with *Irf4* siRNA for 24 h, then treated with IL4 (100 ng/ml) for an additional 24 h, and the cell extracts were harvested for measuring the expression of *Retnla*. Data are representative of three independent experiments and presented as mean ± SEM; Differences between two groups were identified using the Student t-test (**A, D**) and multiple groups (**E**) using one-way ANOVA.

**Figure S4**

**Fig. S4 | Efficiency of mature SREBP2 overexpression.** Mature SREBP2-overexpressing THP-1 cells were harvested for assessment of mature SREBP2 expression by western blotting. Data are representative of three independent experiments.

**Figure S5**

**Fig. S5 | The flow cytometry gating strategies for RETNLA^+^ IMs and Th2 cells.** **A, B** Representative flow cytometry plots showing gating for RETNLA^+^ IMs (live SSC^low^CD45^+^CD11c^-^F4/80^+^Ly6c^-^CD64^+^, **A**) and Th2 cells (live SSC^low^CD3^+^CD4^+^ICOS^+^ST2^+^, **B**) in lung tissues.

**Supplementary tables**

**Table S1. Primers used for genotyping**

| Genes | Primer sequence (5’-3’) |
| --- | --- |
| *Scap* | Forward GCTCTGCGCATCCTATCCAATTCCC |
|  | Reverse CAGCCGGCAAGTAACAAGGGATCCG |
| LysMCre | Wild type TTACAGTCGGCCAGGCTGAC |
|  | Mutant CCCAGAAATGCCAGATTACG |

**Table S2. Primers used for Q-PCR**

| Species | Genes | Primer sequence (5’-3’) | | |
| --- | --- | --- | --- | --- |
| Mouse | *Actb* | | Forward AGAGGGAAATCGTGCGTGAC | |
|  |  |  | Reverse CAATAGTGATGACCTGGCCGT | |
| Mouse | *Il4* | | Forward CCCCAGCTAGTTGTCATCCTG | |
|  |  |  | Reverse CAAGTGATTTTTGTCGCATCCG | |
| Mouse | *Il13* | | Forward CCTGGCTCTTGCTTGCCTT | |
|  |  |  | Reverse GGTCTTGTGTGATGTTGCTCA | |
| Mouse | *Arg1* | | Forward TTGGGTGGATGCTCACACTG | |
|  |  |  | Reverse GTACACGATGTCTTTGGCAGA | |
| Mouse | *Retnla* | | Forward CCAATCCAGCTAACTATCCCTCC | |
|  |  |  | Reverse CCAGTCAACGAGTAAGCACAG | |
| Mouse | *Hmgcs1* | | Forward TGAACTGGGTCGAATCCAGC | |
|  |  |  | Reverse CCTGTAGGTCTGGCATTTCCT | |
| Mouse | *Hmgcr* | | Forward TGTTCACCGGCAACAACAAGA | |
|  |  |  | Reverse CCGCGTTATCGTCAGGATGA | |
| Mouse | *Fdps* | | Forward ATGCCATCAACGACGCTCTG | |
|  |  |  | Reverse CCGATCTCTGTCTGATAGGAACT | |
| Mouse | *Sqle* | | Forward GACCTCGTTCGTGACGGAC | |
|  |  |  | Reverse CTCCCCAACTATCCTGTCGG | |
| Mouse | *Cyp51* | | Forward GACAGGAGGCAACTTGCTTTC |  |
|  |  |  | Reverse GTGGACTTTTCGCTCCAGC |  |
| Mouse | *Sc5dl* | | Forward CAGTGCCGCCGATTACTACTT |  |
|  |  |  | Reverse AGGAGGCTAATAGTTTGTCGGAT |  |
| Mouse | *Hsd17b7* | | Forward CCTCTCGCAATGCAAAGAAGG | |
|  |  |  | Reverse GAGGTCGGTAGCATATTTGGAAG | |
| Mouse | *Scap* | | Forward GCTCGTGACCACCTACATCAT | |
|  |  |  | Reverse CCCCACTTGGACTTGACCA | |
| Mouse | *Srebf2* | | Forward GTTGACCACGCTGAAGACAGA |  |
|  |  |  | Reverse CACCAGGGTTGGCACTTGAA |  |
| Mouse | *Irf4* | | Forward TCCGACAGTGGTTGATCGAC |  |
|  |  |  | Reverse CCTCACGATTGTAGTCCTGCTT |  |
